# Role of the Fatty Acid Binding Proteins in Cardiovascular Diseases: A Systematic Review

**DOI:** 10.3390/jcm9113390

**Published:** 2020-10-22

**Authors:** Hien C. Nguyen, Mohammad Qadura, Krishna K. Singh

**Affiliations:** 1 Department of Anatomy and Cell Biology, Schulich School of Medicine and Dentistry, University of Western Ontario, London, ON N6A 5C1, Canada; hnguy29@uwo.ca; 2 Vascular Surgery, Keenan Research Centre for Biomedical Science and Li Ka Shing Knowledge Institute of St. Michael’s Hospital, Toronto, ON M5B 1W8, Canada; mohammad.qadura@utoronto.ca; 3 Medical Biophysics, Schulich School of Medicine and Dentistry, University of Western Ontario, London, ON N6A 5C1, Canada; 4 Pharmacology and Toxicology, University of Toronto, Toronto, ON M5S 1A1, Canada

**Keywords:** fatty acid binding protein, FABP, cardiovascular disease, heart failure, peripheral artery disease, atherosclerosis

## Abstract

Cardiovascular diseases (CVD) remain a global pandemic and leading cause of deaths worldwide. While several guidelines have been developed to control the development of CVDs, its prevalence keeps on increasing until this day. Cardiovascular risk factors, such as reduced exercises and high fat or glucose diets, culminate in the development of the metabolic syndrome and eventually atherosclerosis, which is driven by high blood lipid and cholesterol levels, and by endothelial dysfunction. Late complications of atherosclerosis give rise to serious clinical cardiovascular manifestations such as myocardial infarction and hypertension. Therefore, endothelial functions and the lipid metabolism play critical roles in the pathogenesis of CVDs. Fatty acid-binding proteins are a family of intracellular proteins expressed in many cell types known mainly for their interaction with and trafficking of cellular lipids. The roles of a number of isoforms in this family have been implicated in lipid metabolic homeostasis, but their influence on endothelial function and vascular homeostasis remain largely unknown. This review’s purpose is to update fundamentals about the connection between cardiovascular disease, metabolism, endothelial function, and mainly the roles of fatty acid-binding proteins.

## 1. Introduction

Cardiovascular disease (CVD), remain the number one cause of global deaths, responsible for about 17.5 million deaths worldwide annually [1]. As a vast multitude of cardiovascular risk factors have been identified, while some are genetic dispositions, many risk factors are modifiable and commonly arise under the current economic climate, especially from the commercialized diets and excessively sedentary lifestyles. Acquiring, maintaining, or exacerbating cardiovascular risk factors culminate in conditions of the metabolic syndrome, which include hypercholesterolemia, dyslipidemia, type-II diabetes, as well as endothelial dysfunction and cardiovascular diseases. Endothelial dysfunction and hypercholesterolemia, among other disorders, are the driving mechanisms of atherosclerosis, which is the major cause of CVDs. Indeed, clinical complications of the heart and blood vessels, such as myocardial infarction and peripheral artery disease, account for the majority of the morbidity/mortality associated with the metabolic syndrome. As the body’s lipid levels correlate with cardiovascular risk, fatty acid binding proteins are small intracellular proteins in many cell types responsible for the roles of lipid-trafficking. Many isoforms of this protein family have been identified and described for their shared mechanisms of interacting and binding with cytosolic lipids ligands for their escorts to coordinated sites of metabolism and signaling. However, the unique functions of each isoforms in specific cell types are still largely unknown. Nevertheless, the roles of certain isoforms in cell-types specialized for lipid processing, such as adipocytes, macrophages, and endothelial cells, have been implicated in the regulation of systemic homeostasis, suggesting their importance in the development of metabolic and cardiovascular disorders. In order to strengthen the focus of current research on the fatty acid binding proteins family, we conduct this review to provide the relevant fundamentals on the interconnection between cardiovascular diseases, the metabolic syndrome, and the fatty acid binding proteins family. In addition, as endothelial dysfunction is a key mechanism of atherosclerosis and, hence, an important target for CVDs, the endothelium lining of blood vessels is known for an extensive capacity of vascular homeostasis. This review will also lay out what is currently known about endothelial metabolism and the pathophysiological role of fatty acid binding proteins in endothelial cells.

## 2. Cardiovascular Disease

Cardiovascular disease (CVD) is the disorder of the circulatory system affecting the blood vessels and the vascularized systems, prominently the heart, brain, and limbs. CVDs can be classified based on whether an ischemic condition is involved, wherein the affected blood vessels are luminally thickened leading to compromised blood delivery and ischemia in the downstream organs. Ischemic CVDs are more common and consist of ischemic heart diseases (IHD), cerebrovascular diseases, and peripheral vascular diseases (PAD) referring to the blockages in the arteries supplying the heart, brain, and peripheral extremities, respectively. These conditions are responsible for the serious clinical manifestations such as hypertension, angina (chest pain), acute myocardial infarction (AMI), stroke, or claudication and limb ischemia [2].

### 2.1. The Burden of Cardiovascular Disease

Cardiovascular disease is a leading cause of annual deaths worldwide and in the United States. According to the World Economic Forum, CVD represent 50% of global deaths due to noncommunicable diseases deaths, notably 37% of deaths in the population of less than 70 years of age. The World Health Organization reported an annual CVD death rate of 17 million people, particularly throughout 2016 and 2017, with more than 70% of deaths occurring in low- to middle-income countries. CVD prevalence is higher in males than females but increases in older age groups for both sexes (notably over 35 years of age) [3]. The prevalence is also higher for the population with lower social-economic status [4]. According to a 2020 report from the American Heart Association, the global age-adjusted death rate, incidence, and prevalence of CVD in 2017 was about 233 deaths per 100,000 population, 6000 cases per 100,000, and 485 million cases in total, respectively. These numbers are expected to increase. Notably, Eastern Europe and Central Asia experienced the highest CVD mortality rates, while the prevalence was high in the United States, Central Europe, North Africa, and the Middle East. In the United States, the total cost of CVD in 2014 was approximately $351 billion and $213 billion of this amount was direct cost encompassing prescription medication and medical services such as physicians, professionals, and hospitals. The other $138 billion was indirect cost associating with disability, premature death, and losses of employment, earnings, and productivity. It is estimated that the total costs of CVD are expected to reach $1.1 trillion by 2035. In 2016, about 48% of total American adults were diagnosed with CVDs while over 1000 CVD deaths occurred daily. In 2017, CVD was responsible for the deaths of about 220 per 100,000 population and claimed more lives than cancer and chronic lung diseases combined. By 2030, if it remains a leading global cause of death, CVD is expected to account for over 20 million deaths [5,6].

### 2.2. Risk Factors

Risk factors of CVDs are quite common, and many have been identified and categorized into modifiable and non-modifiable risk factor categories. Modifiable risk factors can be improved or prevented by drug treatments, or by adopting a healthy lifestyle and social changes such as healthy eating, exercise, and smoke cessation. Physical inactivity and poor diets (high in glucose and saturated- or trans-fats) are the major modifiable risk factors for CVDs as they lead to high blood lipids and cholesterols. These conditions are the developing mechanisms of atherosclerosis, which is the most common cause of CVD. Smoking can promote CVDs by damaging the endothelium, the cell-lining of all blood vessels, and the liver, in turn inducing endothelial dysfunction and reducing the production of high-density lipoprotein, which contribute to atherosclerosis and hypercholesterolemia [7,8]. Non-modifiable risk factors dictate that some individuals are intrinsically more vulnerable than others to cardiovascular complications, such risk factors include older ages, male sex, and genetic factors such as family history of CVDs. In aging individuals, especially over age 55, the rise in cardiovascular risk is commonly attributed to increasing blood cholesterols [9] and degrading vascular integrities, such as loss of arterial elasticity and reduced arterial compliance [10]. CVDs diagnosis in men occur 10 years earlier than women on average, and the incidence rates for women is substantially lower compared to men of the same age or postmenopausal women [11]. Explanations provided for this phenomenon include the female sex hormones, estrogens, which are prominently active in pre-menopausal women and have cardioprotective effects [12]. Women may also have fewer typical CVDs symptoms compared to men, making their diagnosis difficult, which, further contribute to the sex-specific gap [13,14]. Genetic risk factors related to CVDs development have also been identified related to vascular health and blood lipid levels. Prominent genetic factors include inherited hypertension and familial hypercholesterolemia, which can lead atherosclerosis [15]. Other non-modifiable risk factors include ethnicity [16] and socioeconomic status [17].

### 2.3. Causes of CVD

A major cause of ischemic CVDs is atherosclerosis [18], which is a chronic and inflammatory vascular disorder characterized by the narrowing of the blood vessel’s lumen by lipid-laden plaques raised and thickened from the vascular luminal walls. The development of these atherosclerotic plaque can begin since childhood [19] with the initial formation of fatty streaks, which are soft vascular lesions composed of foam cells and lipid deposits [20]. These lesions grow over-time, protruding into the vascular lumen, in turn narrowing the luminal space, restricting blood flow, and ultimately causing ischemia to the vascularized organs [21]. Late-stage atherosclerosis underlies the common ischemic CVDs, such as coronary arteries disease (CAD) and peripheral arteries disease (PAD), which arise from the ischemia of the heart or the lower limbs, respectively. Mechanistically, the development of fatty streaks into the mature atherosclerotic plaques is driven by hypercholesterolemia. Circulating low-density lipoproteins (LDL), or the body’s cholesterols, have the tendency to infiltrate across the endothelium into the blood vessel’s intimal layer. In the intima, where cellular metabolic activities are active, the LDLs can be processed to become oxidized LDLs (oxLDL), at which point they can introduce oxidative stress to a variety of cells in the vascular wall, inducing inflammation. In response, circulating leukocytes, including neutrophils and monocytes, are recruited to the inflamed vessel’s wall. Here, the monocytes differentiate into macrophages and, together with the already presented tissue macrophages, engage in the uptake of oxLDL through scavenging receptors for clearance. Some of these scavenger macrophages become overwhelmed by the introduced oxidative stresses and undergo apoptosis, a process prolongedly stalled by the filling lipids they picked up, at which stage they are referred to as lipid-laden foam cells that become stuck in the damaged vascular wall. Due to hypercholesterolemia, and also to reduced serum levels of the cholesterol-clearing high-density lipoproteins (HDL) [22], excessive presences of oxLDLs in the vascular wall easily overwhelm the scavenging mechanism, induce apoptosis, and produce foam cells population at a massive level. Early signs of concentrating foam cells in the intimal layer indicates the formation of soft lesion fatty streaks. Overtime, the increasingly accumulating foam cells build up the lipid core of the atherosclerotic plaque. In maturing the plaque, high levels of intimal oxLDLs also stimulate the sub-intimal vascular smooth muscle cells to migrate up to the subendothelial layer and proliferate, forming a fibrous structure covering the lipid cores, which thickens the plaque overtime. The developing of the lipid core and the fibrous cover chronically raise and mature the atherosclerotic plaque. Late-developing atherosclerotic plaques, already narrowing the vessel’s lumen, are also susceptible to rupturing, upon which thrombosis is induced and followed by the complete blockage of the vessel’s lumen, leading to the direct ischemia to downstream vascularized organs. The formed clot itself may induce thromboembolism, in which pieces of the clots break and circulate with high risk of lodging in, in turn compromising, other blood vessels [21].

### 2.4. CVD and The Metabolic Syndrome

Many risk factors of CVDs culminate to conditions of the metabolic syndrome that contribute to the development of atherosclerosis. Metabolic syndrome refers to a cluster of metabolic disorders, including hyperglycemia, insulin resistance, and obesity characterized by dyslipidemia and hypercholesterolemia. Individuals with exacerbating conditions of the metabolic syndrome are at risk for obesity, type II diabetes (T2DM), and eventually CVDs among other complications [23].

T2DM and obesity are the metabolic disorders featuring high blood glucose levels and insulin resistance, and dyslipidemia and hypercholesterolemia, respectively. Insulin resistance refers to the unresponsiveness of most cells in the body to insulin, a hormone produced by the beta-cells islets of the pancreas to signals the uptake of blood glucose by cells of various body systems. Many studies have elucidated several mechanisms of obesity that lead to, and exacerbate, the events of insulin resistances, which in turn promote hyperglycemia and T2DM. For instance, T2DM is quite common in patients with obesity, and insulin resistance is strongly associated with elevated blood lipids, which contribute to dyslipidemia featured in obesity [24]. Individuals with both T2DM and obesity are at high risk for CVDs, as the most common deaths with the metabolic syndrome are cardiovascular. For examples, hypertension is very common among patients with T2DM [25].

## 3. The Endothelium and Its Roles in Cardiovascular Disease

### 3.1. The Endothelium

The inner luminal walls of all blood vessels in the circulatory system are lined by a specialized simple squamous epithelium called the endothelium. As it lines the luminal wall of vasculatures, the endothelium is in direct contact with blood and all circulating biomolecules and cells. For this reason, the endothelium was initially thought to only serves as a barrier preventing the access of the blood cells to the vascular matrix. Now, this barrier function is recognized to also mediate selective exchanges between tissues and blood components crucial for body metabolism and paracrinal and endocrinal signaling. The endothelium is also known for its predominant roles in the regulation of vascular tone and compliances, inflammation, wound healing, thrombosis, and angiogenesis [26]. Like other epithelia, the endothelium is attached to a thin basal lamina composed of connective proteins, pericytes, and vascular smooth muscle cells. Together with this scaffolding layer, the endothelium constitutes the intimal layer of all blood vessels. In addition to anchoring the endothelial cells (ECs), components of the basal lamina are regulated, mostly by ECs, to carry out angiogenesis and cell migration; these processes are essentials in development, as well as wound healing and cancer [27].

ECs can produce virtually all the proteins of the basal lamina, including the enzymes involved in remodeling, such as matrix metalloproteinases [28]. There are roughly 35 trillion ECs in a human body occupying an average surface area of 650 m square, which strongly indicates the endothelium’s role in exchange between tissue and blood [29]. ECs are generally squamous, but notably slightly elongated and oriented along the axis of the vessels, such that the shear stresses from flowing blood are minimized. Their morphology, in fact, vary across the vascular tree within the following dimensions: 50–70 μm long, 10–30 μm wide, and 0.1–10 μm thick. Likewise, the functional aspects of the endothelium also vary in degree among different parts of the vascular trees [30]. For example, fenestration is an endothelial feature highlighting the structural and function heterogeneities of the endothelium. More fenestrated endothelia are found in the capillaries of sites with high activities of blood–tissue exchange, such as the renal glomeruli, liver, and spleen, where blood filtration is the primary function. In contrast, ECs of the endothelia lining the blood–brain- or blood–testis-barrier are not fenestrated and rather tightly interconnected and highly impermeable, maintaining a highly selective exchange between blood and tissues [31].

### 3.2. Role of Endothelium in CVD

Physiologically, ECs maintains a non-thrombogenic and non-inflammatory blood–tissue interface with regulated selective permeability. Upon stressful physical, chemical, or infectious stimuli, the injured ECs become activated toward a state of increased permeability, pro-inflammation, thrombosis, and vasoconstriction—a process referred to as endothelial activation, which notably encompasses vasoconstriction localize blood to the injured site; the endothelium become permeable (and adhesive) to leukocytes; ECs change shapes and secrete cytokines to initiate inflammation; as well as growth factors and anti-/pro-coagulant factors to induce and mediate angiogenesis, thrombosis, EC proliferation, and migration in wound healing [32]. Stimuli that activate ECs include hemodynamic or oxidative stresses, advanced glycation end products, or infectious pathogens, as well as metabolic insults, such as hypoxia. If the stimuli alleviate, endothelial activation is reversible, and the physiological resting state of ECs will eventually return. Frequent or prolonged exposure to the stressful stimuli, however, can result in endothelial dysfunction [33].

Endothelial dysfunction, in addition to hypercholesterolemia, is the key mechanism behind atherosclerosis and a wide range of CVDs [34]. Moreover, risk factors of CVDs, including obesity, smoking and alcohol abuse, hypercholesterolemia, hyperglycemia, and hypertension have been shown to influence endothelial dysfunction [35]. Endothelial dysfunction is defined as the functional impairment of ECs in regulating vascular homeostasis and characterized by several aspects at the levels of activated ECs: pro-inflammatory; proliferative; hypercoagulability; enhanced apoptosis and presences of free radicals; impaired barrier or increased permeability; impaired endothelium-dependent vasodilation; and dysregulated production and activities of vasoactive factors, including reduced bioactivities of the endothelial-derived nitric-oxide (NO) and enhanced that of vasoconstrictors, such as endothelin-1 and angiotensin II, which are mediators of hypertension [36]. In atherosclerosis, endothelial dysfunction is induced by oxLDL and exacerbates plaque formation and development through several aspects: (1) impaired endothelial barrier function allowing easy LDL penetration into the vessel’s wall; (2) dysregulated endothelial nitric oxide (NO) production reduce vasodilation and increase the production of reactive oxygen species (ROS), which slows down blood flow in favors of thrombosis, and contributes to LDL oxidization, respectively; and (3) injured and activated endothelial cells upregulate pro-inflammatory and pro-coagulative markers, such as intercellular adhesion molecule-I (ICAM-1) and von Willebrand factor (vWF), facilitating leukocytes and platelets adhesion, respectively [37].

### 3.3. Endothelial Fatty Acids Metabolism

Fatty acids (FAs) typically serve as a reliable source of long-term energy in many cells. In some cell-types, such as cardiomyocytes, they are the primary source of energy. In others, such as skeletal muscle cells, FAs are used as the backup energy in glucose deprivation scenarios. When not in use, intracellular FAs are stored in cytosolic lipid droplets. Upon mobilization, they are liberated from these droplets and subjected to fatty acid oxidation (FAO) in the mitochondria to produce acetyl-CoA that can fuel the tricarboxylic acid (TCA) cycle and produce ATPs. Other sources of acetyl-CoAs include glucose and amino acids such as glutamine [38]. In ECs, FAs can be synthesized de novo by FA synthase [39] despite the cell’s capability to uptake them from circulating lipoproteins [40]. Endothelial FAs are notably subjected to the mitochondrial FAO and TCA cycles primarily for the production the intermediates oxaloacetate and α-ketoglutarate, from which aspartate and glutamate are derived to be used in deoxyribonucleotides synthesis and support EC proliferation [41]. In this process, carnitine palmitoyltransferase 1a (CPT1a), which imports FAs into the mitochondria, was shown to be a rate-controlling enzyme of endothelial FAO that selectively stimulates EC proliferation. Inhibition or silencing of CPT1a resulted in decreased deoxyribonucleotide triphosphate (dNTP) pool and impaired sprouting, which relies on EC proliferation in vitro. Indeed, the retinal vascular network in mice deficient for endothelial-specific CPT1 were compromised for branch points number and radial expansion. However, the migratory property of the ECs in these mice remain normal [41]. Compromising endothelial CPT1 also reduced FAO and increased endothelial permeability [42]. Moreover, FAO was demonstrated to be essential in EC specialization by inhibiting endothelial-to-mesenchymal transition through transforming growth factor β [43]. In addition to serving as lipid storages, the endothelial lipid droplets were also shown to protect against endoplasmic reticulum stress [38]. Mobilized FAs in ECs are also utilized for modulating the EC membranes lipid composition [44], as well as producing lipid-derived arachidonic acid metabolites, such as prostacyclins, which are regulators of endothelial inflammation, coagulation, and vascular homeostasis [45].

## 4. Fatty Acid Binding Proteins

Lipids are vital components of many biological processes and crucial in the pathogenesis of numerous common diseases, but the specific mechanisms coupling intracellular lipids to biological targets and signalling pathways are not well understood. This is particularly the case for cells burdened with high lipid storage, trafficking and signalling capacity such as adipocytes and macrophages. Here, we discuss the central role of lipid chaperones—the fatty acid-binding proteins (FABPs)—in lipid-mediated biological processes and systemic metabolic homeostasis through the regulation of diverse lipid signals, and highlight their therapeutic significance. Pharmacological agents that modify FABP function may provide tissue-specific or cell-type-specific control of lipid signalling pathways, inflammatory responses and metabolic regulation, potentially providing a new class of drugs for diseases such as obesity, diabetes and atherosclerosis.

The body’s lipids are physiologically essential. In addition to serving as effective long-term metabolic energy storages, cellular lipids can have signaling roles in many metabolic and inflammatory pathways. For instances, the eicosanoids, such as prostaglandins, are derived from fatty-acids metabolism and mediate the acute inflammatory responses [46]. In addition, lipid levels in adipocytes dictate their production of cytokines and adipokines, such as leptin and adiponectin, that have potent impact on inflammation and metabolism [47]. Pathologically, as the metabolic syndrome is closely linked to cardiovascular risk, diverse lipid signals and cells with high capacity of lipids storage, trafficking and signaling, such as adipocytes and macrophages, are crucial in the pathogenesis of CVDs. Therefore, lipids-related physiology and cardiovascular impacts crucially depend upon the specific processing and management of the bioavailability of cellular lipids. Such roles have been described for the prominently expressed fatty acid binding proteins (FABPs), lipid-chaperones that regulate many lipid-related processes. The functional aspects of the FABPs are currently being investigated to provide pharmacological or diagnostic targets for controlling the body’s lipid signaling and the associated inflammatory and metabolic mechanisms, which helps develop treatment for atherosclerosis and the metabolic syndrome.

The FABPs are small (12–15 kDa) cytosolic proteins abundantly expressed in tissues with active lipid metabolism, such as the heart and liver, or cell types specialized for lipid storage, trafficking and signaling, such as adipocytes and macrophages [48]. They are a multigene family, well-conserved, and known to be central in a variety of metabolic and cardiovascular disorders, including obesity, diabetes and atherosclerosis [49]. Structurally, all members of the FABP family share a β-barrel signature that consists of a water-filled cavity and a site that binds specific lipid-ligand unique for each member [50]. Nine members of the FABP family have been identified (FABP 1–9) with 20–70% sequence homology among members and each named according to the most abundantly expressing tissue in addition to their designed number. For instance, FABP3 is also heart-type FABP which is most abundant in cardiomyocytes; FABP4 and 5 are adipocyte- and epidermal-type and expressed most prominently in the respective tissues [51]. Despite the unique tissue-expression pattern of each member, in general, tissues with active lipid metabolism tend to express FABPs and, often, more than one isoform. For instance, the small intestine where active absorption of diet lipid take place express prominently FABP2, but also FABP1 (liver FABP) and FABP6 (ileal FABP) [52].

Functionally, the FABPs are known to reversibly interact with hydrophobic ligands with various affinities and mediate their escorts to coordinated sites of lipid metabolisms or signaling, typically serving as intracellular lipid chaperones. Reports up-to-date have documented some of the targeted sites to be lipid droplets for storage, plasma membrane in lipid import and export, mitochondria for lipid-metabolism, as well as the endoplasmic reticulum for phospholipid biosynthesis, specific enzymes for the production of lipid-derived signaling molecules, and the nucleus where their physical interaction with the peroxisome proliferator-activated receptors have been reported [53]. For instance, FABP1 was shown to regulate PPARα in mammalian renal COS-7 cells [54]. However, the promoter of the FABP1 gene itself contains a peroxisome-proliferator response element and, accordingly, FABP1 transcript level was shown to be regulated by intracellular fatty acids, dicarboxylic acids and retinoic acid [55]. The degree of expression may reflect the lipid-metabolizing capacity of a given tissue or cell type and can be modulated upon changes in the bioavailability of lipids, as in lipid exposure or usage processes ([56]). While there is a strong regulatory connection between FABP expression and lipid related signaling, the exact function of different FABPs member remain poorly understood as their general mechanism is associated with a vast scope of complex lipid-related regulatory pathways. For example, FABP2s are abundantly expressed in the small intestine and known to bind absorbed saturated long-chain fatty acids with a high affinity. The lipid-intracellular trafficking of FABP2 is thought to be within the lipid-uptake, lipid-sensor, and lipoprotein synthesis pathways. However, complete ablation for FABP2 in mice did not compromise fat absorption but resulted in larger livers and higher triglyceride levels in males and the opposite in females [57]. In another example, studies have shown epidermal FABP5 influencing cell-survival pathways through PPAR-δ [58]. As the exact functions of each unique FABP members are still under investigation, large resources and attention in metabolic and cardiovascular research are being directed at three members among others: FABP3, 4, and 5.

### 4.1. FABP3

FABP3 (heart-FABP) is expressed most abundantly in myocardiocytes and skeletal muscle. As lipid chaperone, myocardial FABP3 is essential for the metabolic homeostasis of cardiac function. Physiologically, 70% of the energy in the heart are derived from the oxidation of fatty acids within the mitochondria and peroxisomes, which require lipid-trafficking mechanisms [59]. Increased exposures to fatty-acids were shown to upregulate H-FABP in myocytes [60]. FABP3 is also influenced by the metabolic essential PPAR-α agonists [61]. Diabetic patients, within whom fatty acids become the primary source of energy, also exhibited upregulated cardiac FABP3 [62]. In addition, FABP3-deficient mice showed elevated plasma free fatty acids and were compromised for cardiac fatty acid uptake, resulting in reduced exercise tolerance and a switch toward rapid glucose usage in the heart that leads to cardiac hypertrophy [63]. Meanwhile, similarly to other FABP members, FABP3 was also found in a multitude of other tissues to a lesser extent, brain, testis, kidneys, adrenal glands, and others [50]. For this notion, the unique function of FABP3 remains complex and unclear. For instance, while the FABP3 in skeletal muscles mediate the uptake and escorting of fatty acids to the mitochondrial oxidation system as similar as cardiac FABP3, increased apoptosis and exacerbated cardiac dysfunction has been associated with FABP3 overexpression in myocardiocytes [64].

Despite the functional complexity, FABP3 is currently utilized as a clinical biomarker for cardiac injury and heart failure, particularly in the diagnosis of myocardial infarction (MI). MI is characterized by the death of heart tissues from injuries commonly due to atherosclerotic-mediated cardiac ischemia. The current diagnostic approaches for MI include assessing initial chest pain, characteristic electrocardiography, and the detection of biomarkers for myocardial injury [65]. During a heart injury, myocardiocytes suffer a breakdown of cellular and subcellular components, which is followed by the releases of these biomolecules from the injured cells into the circulatory system. Of clinical significance is the leakage of the cytosolic myocardial proteins; their releases serve as a pathological biomarker that can be detected and measured in blood to enable early and efficient clinical assessment of cardiac injury. The more effective biomarkers are more cardiac specific and more abundantly expressed in the myocardiocytes. Clinical guidelines for MI dictates that detecting/excluding MI within the first 6 h of chest pain would bring about the most effective clinical responses [66]. Currently, the only gold-standard biomarker for MI is the cardiac troponin, particularly the cardiac-specific subunits I and T, which can be detected 2–4 h after the onset of chest pain [67]. FABP3 has been proposed as an effective biomarker of myocardial injury. Under normal conditions, the cytosolic to plasma presence ratio of myocardial FABP3 is significantly high, with negligible plasma concentration of FABP3 [68]. Within 30 min of chest pain, blood FABP3 begins to rise and peak in a few hours before returning to baseline via renal clearance in about 24 h. This early release of FABP3 from injured myocardium has been observed in both animal models [69] and MI patients [70]. Recently our research group has demonstrated that patients with PAD have elevated plasma levels of FABP3. Our data demonstrated that the circulating levels of FABP3 increase as the severity of PAD worsens. However, extensive research is still required to demonstrated utilizing FABP3 as a biomarker for PAD [71].

### 4.2. FABP4 and 5

FABP4 (adipose-FABP) have been described for their roles in the development of the metabolic syndrome through their mechanisms in adipocytes as well as macrophages. Both the differentiation of functional adipocytes [72] and macrophages [73] involved the regulation of FABP4, and cellular signals regulating FABP4 in adipocytes and macrophages include fatty acids, agonists of PPAR-γ, insulin, lipopolysaccharide, and oxLDL [74]. Reduced lipolysis efficiency was observed in adipocyte-specific-FABP4-deficient mice [75], and these mice were also found with ameliorated insulin resistance during diet-induced obesity ([76,77]). Moreover, apolipoprotein-E-deficient mice with FABP4 deficiency were found to be protected against atherosclerosis with or without induction by high-cholesterol Western diets [78], but how FABP4 deficiency can alter insulin resistance and lipid metabolism remain to be revealed. FABP4 was also shown to be released from adipocytes into blood. While their biological roles in blood remains unknown, serum FABP4 has been suggested as a potential biomarker for metabolic syndrome and CVDs [79]. In macrophages, FABP4 was found to modulate inflammation and cholesterol concentration. Administration of the cholesterol-lowering atorvastatin was found to suppress FABP4 expression in macrophages in vitro [80]. In addition, enhanced cholesterol efflux was observed in macrophages with elevated PPAR-γ activity from FABP4-deficient mice from a study that demonstrated macrophage’s FABP4 playing a role in foam-cell formation through regulating the PPAR-γ–liver X receptor-α (LXR-α)–ATP-binding cassette A1 (ABCA1) pathway. The same study also found suppressed production of cytokines and pro-inflammatory enzymes, such as TNF-α and COX2, respectively in macrophages of mice deficient for FABP4 [81].

The epidermal-type FABP5 is most prominently expressed in the skin cells, but it is also expressed in multiple other tissues, including adipocytes and macrophages, as well as tongue, brain, kidney, liver, lung, and testis [74]. In adipocytes, FABP5 expression is significantly minimal compared to FABP4 [82], but loss of FABP4 induce the upregulation of FABP5 that, in fact, masked the phenotypic effects of FABP4-deficiency [76]. Unsurprisingly, FABP4 compensatory upregulation is not observed in adipocytes of FABP5 deficient mice due to the already higher presence of FABP4 in adipocytes [49]. In macrophages, the expression ratio of FABP4 and 5 is about identical, but no compensatory FABP5 expression is observed in FABP4-deficient mice [83]. Due to this wide and complicate pattern of tissue expression and regulation, the unique function of FABP5 remains unclear. Nevertheless, several in vivo phenotypes regarding FABP5 expression are relevance to the metabolic syndrome. Transgenic mice overexpressing adipose-specific FABP5 exhibited enhanced lipolysis [84] and reduced insulin sensitivity [49]. On the other hand, increased insulin sensitivity was observed in adipocytes from FABP5 deficient mice [49]. FABP5 deficient mice also appeared healthy, without any changes in the normal epidermal fatty-acid composition [85].

As macrophages accumulation in adipose tissues characterize the enhanced inflammatory response and risks for insulin resistance and CVDs in obesity [86], the roles of FABP4 and 5 in both adipocytes and macrophages contribute to the inflammatory and metabolic aspects of the metabolic syndrome and atherosclerosis [48]. In general, adipocytes and macrophages in mice deficient for these FABPs were more insulin sensitive. Obese mice deficient for both FABP4 and 5 exhibited reduced tissue fatty-acid composition and did not develop insulin resistance [87]. Even when the ApoE^-/-^ model was integrated, these mice showed less atherosclerosis development and increased survival compared to wild-type and either the individual FABP-deficient counterparts [88]. The mice deficient for FABP4 and/or FABP5 also exhibited increased fatty acids levels in plasma [89], suggesting that the intracellular bioavailability of lipids, rather than the total body’s amount, is more relevant to the development of metabolic and cardiovascular disorders. Recently, we were able to demonstrate that FABP4 levels were elevated in diabetic patients with PAD [90]. The elevation in FABP4 was independent of confounding factors such as age, sex, or prior history of CAD. Therefore, our work raises the possibility of utilizing FABP4 as a biomarker for diagnosing PAD in diabetic patients.

### 4.3. FABP in Endothelium

Although glucose is the primary source of energy in endothelium [91], ECs have been demonstrated for extensive lipid-processing mechanisms, including lipid uptakes and transport, FA metabolisms pathways for the synthesis of deoxyribonucleotide triphosphate fueling proliferation and lipid-derived arachidonic acid metabolites. Moreover, cholesterols metabolism was also shown in ECs for their expression of the Niemann–Pick disease type C (NPC) 1 and NPC2 proteins, which mediate cholesterol uptake and trafficking [92]. The activities of endothelial mammalian target of rapamycin (mTOR), which is central player of a signaling network regulating cell growth and proliferation, is dependent on intracellular cholesterol trafficking. Pharmacological blockade of cholesterol trafficking by itraconazole or by silencing NPC 1 and 2 led to inhibition of mTOR activity in ECs [92]. These observations suggest ECs to be active sources of lipid signaling and metabolism. However, few studies have been able to demonstrate the expression and activities of FABP in the endothelium. Nevertheless, two FABP members have been identified in ECs. The expression of FABP3 and FABP5 have been described in the microvascular of cardiac tissues and skeletal muscles. In addition, FABP4 and FABP5 expressions were found in the microvasculars of other organs with active fatty acids metabolism, including the liver and adipose tissues [93,94]. Moreover, Masouyé et al. presented that ECs are capable of fluctuating their expression of FABPs depending on environmental factors, such as between tissue and culture conditions. This study detected FABP5 by immunohistochemistry in cultured human umbilical vein ECs (HUVECs), but not in the endothelium of the umbilical vein from which the HUVECs were isolated [95].

## 5. Closing Remarks

The endothelium demonstrates a remarkable capacity of lipid signaling and metabolism, which appears to be linked to several aspects of endothelial functions, including the regulation of endothelial membrane integrity, angiogenesis, inflammation, coagulation, and vascular homeostasis. However, very few studies have described clearly the functions of FABPs whether in general or in the specific expressing tissues such as the endothelium. Nevertheless, the roles of FABPs are highly relevant to CVD research. For instance, while FABP3 is an effective biomarker of cardiac injury, our recent work on patients with peripheral artery disease (PAD) and critical limb ischemia, which are common complication of atherosclerosis, showed elevated blood FABP3 in association with PAD’s severity in the absence of any signs of myocardial ischemia. These patients were also negative for the gold-standard cardiac injury biomarker Troponin T (unpublished data). This suggests a possible rise of blood FABP3 independent of myocardial injury and, while the source of this FABP3 release is not known, we suspect ECs for their close connection between endothelial dysfunction in atherosclerosis. Additionally, we evaluated plasma FABP4 in diabetic patients with PAD in another recent study and observed an over twofold increase in plasma FABP4 levels in diabetic patients with PAD compared to diabetic patients without PAD [90]. We also found a direct relationship between increasing FABP4 levels and worsening PAD severity in the same study [90]. Moreover, our lab preliminarily demonstrated the basal expression of not only FABP3 but also FABP4 and FABP5 in HUVECs. Unsurprisingly, how FABP3, FABP4, and FABP5 function uniquely in ECs remain to be elucidated. For future goals, the roles of FABPs in ECs can be elucidated in vitro through loss- and gain-of-function schemes (e.g., using silencing RNAs or overexpressing adenovirus) with wet-lab analyses and assays for the relevant protein factors and aspects of endothelial functions. Investigations on the interacting partners and mechanisms, or specific pathways that the FABPs influence in ECs also leave much to be desired, calling for gene array technology. Endothelial FABPs should be demonstrated in several in vitro EC models, such as the standard HUVECs and more specialized human coronary artery endothelial cells (HCAECs) to account for the environmental conditions potentially causing differential expression of FABPs in ECs. In vivo, atherosclerotic mice null for Apo-E with endothelial-specific loss of FABP targets could be chosen to study endothelial-specific FABPs behaviors. Overall, as endothelial dysfunction is the key developing mechanism of atherosclerosis and CVDs, the FABPs and their lipid trafficking mechanisms have been demonstrated for their association in many crucial aspects of cardiovascular complications, including inflammation, hyperlipidemia, and insulin resistance. Therefore, further investigations on the pathophysiological roles of FABPs in the endothelium are warranted. The fundamental findings of such endeavors hold promises for therapeutics and clinical strategies seeking to control lipid metabolism and endothelial dysfunction at the level of the endothelium.
